# The effect of value on long-term associative memory

**DOI:** 10.1177/17470218211014439

**Published:** 2021-05-15

**Authors:** Xiaotong Yin, Jelena Havelka, Richard J Allen

**Affiliations:** School of Psychology, University of Leeds, Leeds, UK

**Keywords:** Long-term memory, associative memory, value, prioritisation/prioritisation, delay

## Abstract

Items with high value are often remembered better than those with low value. It is not clear, however, whether this value effect extends to the binding of associative details (e.g., word colour) in episodic memory. Here, we explored whether value enhances memory for associative information in two different scenarios that might support a more effective process of binding between identity and colour. Experiment 1 examined incidental binding between item and colour using coloured images of familiar objects, whereas Experiment 2 examined intentional learning of word colour. In both experiments, increasing value led to improvements in memory for both item and colour, and these effects persisted after approximately 24 hr. Experiment 3a and Experiment 3b replicated the value effect on intentional word–colour memory from Experiment 2 while also demonstrating this effect to be less reliable when word colour is incidental to the encoding phase. Thus, value-directed prioritisation can facilitate episodic associative memory when conditions for binding are optimised through the use of appropriate to-be remembered materials and encoding conditions.

In everyday life, we are often presented with a large amount of information, often varying in importance or goal-relevance. As such, being able to selectively prioritise more valuable information is of great utility in the context of limited capacity memory and attentional systems. A growing body of research suggests that assigning high point value to some items can give them priority for retention and retrieval in working memory (Hitch et al., 2018; Hu et al., 2014, 2016), and in long-term memory (LTM; Castel et al., 2002, 2013). Studies using the Remember/Know (RK) paradigm further indicate that value improves LTM by selectively enhancing R responses, which indicate a conscious recollection of associative information from an episode (Elliott & Brewer, 2019; Hennessee et al., 2017, 2018).

However, inconsistent results have been observed regarding value effects on associative memory. Some studies found that higher-value items were associated with better associative memory, such as item-location memory (Siegel & Castel, 2018a, 2018b), memory for word pairs (Ariel et al., 2015), and memory for word plurality status (Cohen et al., 2017), whereas others revealed no beneficial effect of value on associative memory, such as memory for the voice gender in which words were presented (Villaseñor et al., 2021) or memory for the colour of visually presented words (Hennessee et al., 2017, 2018). For example, in Hennessee et al. (2017), a series of words were presented in one of four colours, with each stimulus associated with a point-value. Participants were informed that they could earn the point-value associated with the word if they correctly recognised the word at a later test. They were not asked to memorise the point-value or word colour. At test, participants performed an old-new recognition test and for the items they had recognised as old, they indicated the point-value and the colour each word was initially associated with. Memory for high-value words was better than that for low-value words. However, value was not found to affect memory for colour or memory for point-value. When further examining whether associative memory would interact with memory type (recollection or familiarity), Hennessee et al. found that colour memory accuracy was actually lower in high-value recollected items, compared with low-value recollected items.

Although a reduction of associative memory in high-value items may seem counterintuitive, previous studies have revealed behavioural and neural dissociation between memory for item and memory for contextual details (Davachi, 2006; Davachi et al., 2003; Glisky et al., 1995; Old & Naveh-Benjamin, 2008; Spencer & Raz, 1995). In some cases, item memory and associative memory appear to function in a consistent pattern, whereby item memory improves alongside enhanced associative memory. For example, it is well documented that emotional information is often better remembered than neutral information (e.g., Hamann, 2001, for a review). This emotional memory enhancement effect also extends to associative memory, such as memory for visual details of objects (i.e., perceptual features, such as colour, shape, size, and orientation) and memory for colour of words (e.g., Doerksen & Shimamura, 2001; Kensinger & Corkin, 2003; Kensinger et al., 2006). In others, item memory and associative memory act in a tradeoff pattern in which the memory enhancement for item information emerges at the expense of memory for associated details (e.g., Kensinger et al., 2005). For instance, when individuals are confronted with a complex visual scene, memory for the emotional component is enhanced, whereas memory for the peripheral details (e.g., another object nearby or the background of the central object) is reduced (e.g., Kensinger et al., 2005). One possible reason for the different patterns may depend on the effectiveness or strength of binding between item and associative information. In the examples mentioned above, the associative details enhanced together with an item might be categorised as intrinsic features (Godden & Baddeley, 1980). They could be easily integrated and automatically processed when the stimulus is perceived and comprehended. In contrast, conditions eliciting a tradeoff pattern may involve extrinsic features, which are irrelevant to the processing of the stimulus itself and thus more likely to be omitted from further encoding.

By this view, the effects of value on associative memory in previous studies could depend on the type of association being studied and the instructions provided to participants. Positive associative memory value effects may indicate more effective binding between item and associative information, either due to the type of features being examined or the explicit instruction to remember both item and associative information (Ariel et al., 2015; Cohen et al., 2017; Siegel & Castel, 2018a, 2018b). In studies which have found no evidence that value affected associative memory, binding of features may be less effective as the task instructions did not emphasise memory for associative information (Hennessee et al., 2017, 2018; Villaseñor et al., 2021). Thus, it is possible that value effects have not been observed on colour memory due to the dissociation of word and word colour under the incidental learning conditions implemented in studies to date (Hennessee et al., 2017, 2018). Although word colour might be classified as an intrinsic feature (e.g., D’Argembeau & der Linden, 2004; Uncapher et al., 2006), studies indicate distinct processing of word and word colour (Brown et al., 2002) and memory for word colour is poor under incidental learning conditions (Park & Mason, 1982; Park & Puglisi, 1985; Uncapher et al., 2006). Indeed, evidence that value enhances visual working memory has typically so far been observed on colour–shape binding measures in which colour information is made an integral part of the item (Allen & Ueno, 2018; Atkinson et al., 2018; Hitch et al., 2018; Hu et al., 2014, 2016; see Hitch et al., 2020 for a review). Therefore, the first goal of this study is to establish whether value will enhance LTM for item colour under types of binding condition where the association between colour and item may be more likely to be encoded and retained in memory.

This study also examined the longevity of any beneficial effects of value that are observed. A common feature of previous studies is that they have employed immediate or short retention intervals (typically 5 min) between the study and the test phase. To our knowledge, no study has investigated the persistence of value effects using point-values over more extended delay periods. This could help us better understand the underlying mechanisms. For example, Murayama and colleagues have suggested that a reward-related (possibly dopaminergic) memory consolidation process operates over longer periods of time, increasing the effects of monetary value on memory performance (e.g., Murayama & Kitagami, 2014; Murayama & Kuhbandner, 2011). Items that are assigned a higher value may also receive more active attentional processing during encoding (Allen, 2019), creating a stronger representation that is less susceptible to loss over time (either through decay or interference) and thus relatively more accessible than low-value items at longer delays. It is not always the case, however, that memory enhancement effects increase in magnitude over time. For example, the superiority of semantic encoding usually diminishes over a 24-hr or longer delay (e.g., McDaniel & Masson, 1977; Morris et al., 1977; Thapar & McDermott, 2001). A second goal of this study was therefore to explore how the effect of value changes over delays of a few minutes, and 24 hr.

Two factors were identified that might influence the binding between item and colour information, and therefore increase the likelihood of value effects emerging on associative as well as item memory. The first of these was the type of item used as a to-be-remembered stimulus set. Images, relative to words, appear to support effective integration with colour information (Park & Puglisi, 1985) and so may offer an effective context in which value may be applied to enhance associative memory. Thus, while previous work on this topic has used words (Hennessee et al., 2017, 2018), Experiment 1 used images as the stimulus set to explore the effect of value on colour memory. Second, the nature of the encoding phase, and whether participants are asked to intentionally encode item–colour associations, is likely to be important. Indeed, previous studies indicate that associative memory was significantly improved when participants were instructed to intentionally remember both the item and the associative information, relative to remembering the item information only (Chalfonte & Johnson, 1996; Hockley & Cristi, 1996; Light & Berger, 1974; Light et al., 1975). Experiment 2 therefore reverted to a word list paradigm as in previous studies (Hennessee et al., 2017, 2018) but explicitly instructed participants to remember word and word colour intentionally. In both experiments, memory was tested twice, with a 5-min short delay and a 24-hr long delay. Finally, the use of two different point values (i.e., 1 point for low value and 10 points for high value) in this study may have made it relatively easier for participants to distinguish between high- and low-value items and enable a more effective focus on high-value items, compared with previous studies (Hennessee et al., 2017, 2018) that used six different point values (i.e., 1, 2, and 3 points for low value and 10, 11, and 12 points for high value). Therefore, Experiment 3a and Experiment 3b (online experiments) sought to replicate the findings of Experiment 2 under intentional word–colour encoding conditions (Experiment 3b), while also confirming whether value effects on word–colour associative memory are indeed less reliable following incidental encoding of colour (Experiment 3a) in the present paradigm.

## Experiment 1

To date, examination of value effects on item–colour memory have focused on words as a stimulus set, with Hennessee et al. (2017) finding no evidence that value can improve memory for colour of words. This may reflect the irrelevance of the colour to the task at the encoding phase, and the possibility that word meaning is more salient and important than its visual appearance. Encoding of visual images, however, might allow the effective integration of item and colour information, meaning that colour is more reliably included as part of the memory representation that is created when participants prioritise high-value items. Indeed, prior research has shown that memory for colour of pictures was substantially better than memory for colour of words (Park & Puglisi, 1985). Experiment 1, therefore, used coloured pictures as to-be-remembered stimuli. We expected to see a memory enhancement for colour from high-value items. It was also of interest whether this effect would change over time. Thus, a short-term delayed test (approximately 5 min after encoding) and a long-term delayed test (approximately 24 hr later) were conducted.

### Method

#### Participants

Thirty undergraduate students (23 females; mean age = 20.7 years; range = 18–27 years) recruited from the University of Leeds participated in this experiment. All participants were native English speakers, and none reported a history of neurological disorders. Participants had normal colour vision, and correct or corrected-to-normal vision. Informed consent was acquired in accordance with the guidelines set by the University of Leeds’s Psychology Ethics Committee (Ethics reference number: PSC-462).

#### Materials

The stimuli were 176 neutral line drawings of daily objects taken from Snodgrass and Vanderwart (1980) and Cycowicz et al. (1997). Eighty-eight of them were randomly selected during the study phase, with half of them paired with a 1-point value and the other half paired with a 10-point value. Each line drawing was filled with one of the four colours: red, yellow, blue, and green. They did not strongly associate with a particular colour. The remaining 88 images were used as foils during the recognition phase. The images assigned to each participant and the point value and colour assigned to each image were randomised for each individual participant.

#### Procedure

**Figure 1.**
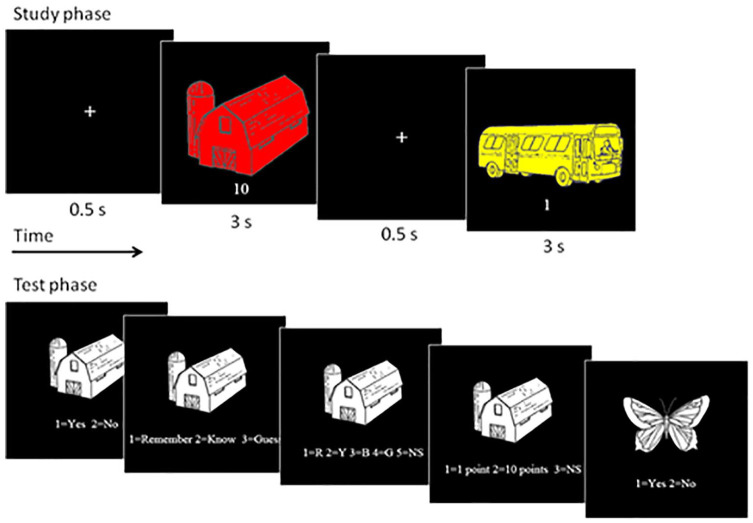
Study and test procedures in Experiment 1.

The experiment consisted of one study phase and two test phases. The study phase and the first test phase were conducted in an experimental lab using PsychoPy 3.0.5 (Peirce, 2007). The second test phase was conducted online using Qualtrics survey software (Qualtrics, 2019) . At study, participants were told that they would be presented with a series of images, each associated with a point-value they could earn later for recognition and their goal was to maximise the score. Participants were not told to memorise the point-value or the word colour. All 88 study images were presented individually for 3 s with a 0.5 s fixation cross interval (see Figure 1). Next, participants completed a brief distractor task (24 simple multiplication and division problems) to reduce mental rehearsal, during a 5-min delay interval. Before completing the recognition test, participants were instructed regarding the difference between remembering (R), knowing (K), and guessing (G) using an adapted form of Gardiner et al. (1998) instructions (see online Supplementary Material A for instructions).

At test, participants viewed a randomised sequence of 88 previously presented images and 88 new images, without colour. They were asked to report whether or not they had seen each of them (1 = Yes, 2 = No). If they chose “Yes,” they were asked to further make an R, K, G judgement (1 = Remember, 2 = Know, 3 = Guess) and report the colour (1 = Red, 2 = Yellow, 3 = Blue, 4 = Green, 5 = Not Sure) and the point-value (1 = 1 point, 2 = 10 points, 3 = Not Sure) of the item; if they chose “No,” no further judgements were required for this image. The next image then appeared and the cycle was repeated (see Figure 1 for an example). The “not sure” option is offered to reduce potential contamination by guessing on the associative memory, as has been implemented in previous studies (e.g., Duarte et al., 2008; Gottlieb et al., 2010; Morcom et al., 2007). All responses were self-paced. Participants were informed that after approximately 24 hr, they would be emailed a link for the second part of the study (with no mention of the retest). Twenty-two hours after participating in the experiment, participants received the link and were asked to complete this phase within 4 hr. The test procedure and the foil set were the same as in the short-delay test, with the exception that the items were presented in a different order relative to the short-delay test, though the order was the same for all the participants at the long-delay test. Participants were asked to complete the test in a quiet area with minimal distractions.

### Data analysis

The primary outcome variable of interest in this experimental series was the accuracy of item–colour memory judgements. However, we also report item recognition memory, RKG judgements, and point memory accuracy to provide a more comprehensive overview of memory performance. Generalised linear mixed effects models (GLMM) were fitted to the data by taking participants and items as random factors. GLMM can estimate variance from overall differences among participants and items (random intercept); it can also estimate variance in their sensitivity to the experimental manipulations (random slope), the latter of which would not be achievable when conducting the classic analysis of variance (ANOVA; although outcomes from these analyses were generally consistent when using repeated measures ANOVA). We fitted GLMM with binomial distribution and logistic link function using the afex package in R (R Core Team, 2020; Singmann et al., 2021), beginning with the maximal model (Barr et al. 2013). The sum-to-zero coding scheme was used for the categorical predictors. To deal with convergence issues, the optimiser was set to “bobyqa” and the derivative calculation was switched off. To deal with singular fit in a model, a step-wise model simplification procedure was performed by dropping the random effects whose variances were estimated as zero (or very small) or correlations were estimated as ±1, and by dropping higher-order random effects. Finally, likelihood ratio tests were performed to confirm that the simplified model was not significantly different from the maximal model. For any observed interactions, the emmeans package (Lenth, 2020) was used to conduct pairwise comparisons (Bonferroni). Separate models were set up for item memory, colour memory, and point-value memory. With regard to RKG responses (ordinal data), a cumulative link mixed model (CLMM) was fitted to the data using the clmm function within the ordinal package (Christensen, 2020). The *p* values for the fixed effects in CLMM were obtained via the Anova.clmm function from the RVAideMemoire package (Hervé, 2021). Analysis of item memory was based on old items; analyses of RKG responses, colour memory, and point-value memory were based on correctly recognised items.

### Results

Most participants completed the 24-hr delayed test within an acceptable time frame (with one night’s sleep; *N* = 23, mean time = 25 hr 3 min, range = 21 hr 58 min–31 hr 31 min). Three participants failed to complete the test. Four participants took more than 2 days to complete the test (i.e., 50h24m, 71h9m, 193h21m, 202h48m), but their memory patterns were similar to the others and including their data does not change the results, so analyses were based on data from 27 participants. In each model, fixed factors included value, retention interval, and their interaction. In the models of item memory and point-value memory, random factors included random intercept and random slope of value and retention interval within participants, and random slope of value within items with intercept. The model of RKG responses has the maximal random effects structure. In the model of colour memory, random factors included random intercept and random slope of value within participants, and within items.

#### Item memory and RKG responses

Mean hit rates; false alarm rates; R, K, and G responses as a function of value, and retention interval are displayed in Table 1. GLMM revealed a main effect of value on item memory, χ^2^(1) = 30.14, *p* < .001, whereby memory for high-value items was better than memory for low-value items. The effect of retention interval was also significant, χ^2^(1) = 8.11, *p* < .01, such that memory was better at the short delay than the long delay. The interaction between value and retention interval was not significant, χ^2^(1) = 1.60, *p* = .21. On RKG responses, there was a main effect of value, χ^2^(1) = 18.30, *p* < .001, with better memory quality for high-value items than low-value items. A main effect of retention interval also emerged, χ^2^(1) = 7.67, *p* < .01, with better memory quality at the short delay than the long delay. There was no interaction between value and retention interval, χ^2^(1) = 0.00, *p* = .99.

**Table 1.** Mean Hit Rates, False Alarm (FA) Rates, Remember (R), Know (K), Guess (G) responses and point-value memory as a function of value and retention interval in Experiment 1.

	Short delay		Long delay	
	High value	Low value	High value	Low value
Hit rates	0.73(0.03)	0.53(0.03)	0.64(0.03)	0.47(0.03)
FA rates	0.09(0.01)		0.20(0.03)	
R	0.54(0.05)	0.45(0.04)	0.46(0.05)	0.36(0.04)
K	0.33(0.04)	0.33(0.04)	0.38(0.04)	0.42(0.04)
G	0.13(0.03)	0.22(0.03)	0.15(0.03)	0.22(0.03)
Point-value	0.51(0.04)	0.52(0.05)	0.46(0.04)	0.36(0.05)

FA: false alarm. Standard errors presented in parentheses.

#### Associative memory

**Figure 2.**
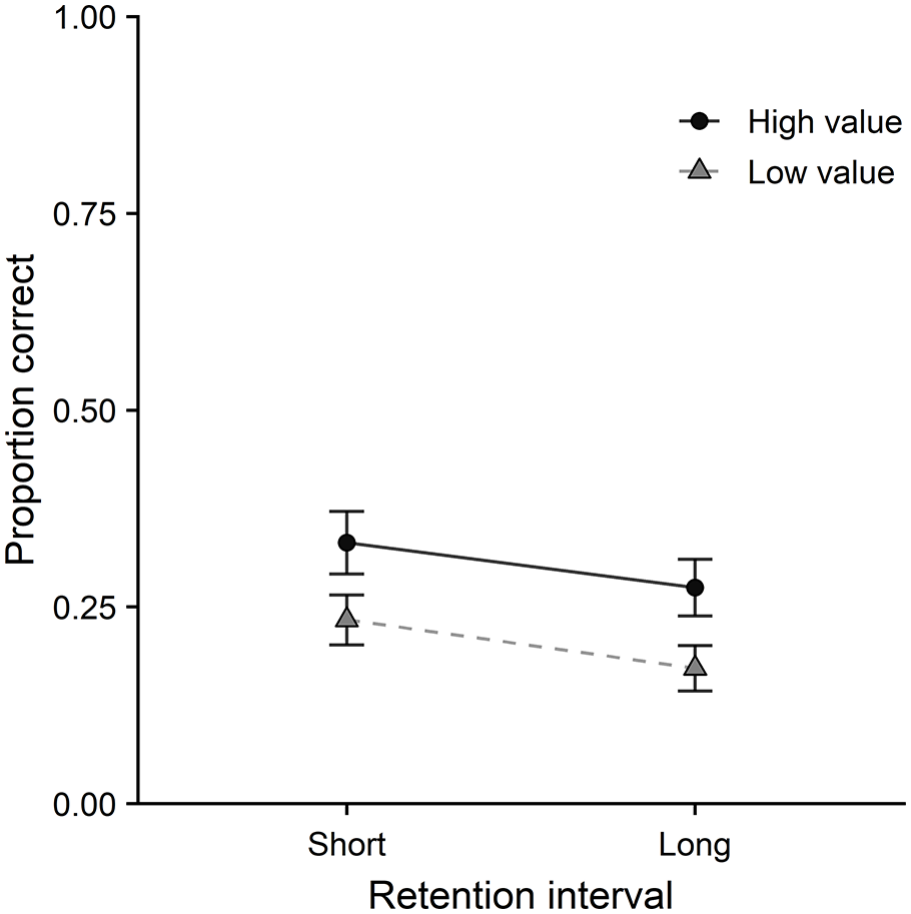
Colour memory performance as a function of value and retention interval in Experiment 1. Error bars represent one standard error of the mean.

Colour memory performance is displayed in Figure 2, as a function of value and retention interval. On colour memory, a main effect of value was observed, χ^2^(1) = 11.89, *p* < .001, with higher accuracy for high- than low-value items. A main effect of retention interval was also observed, χ^2^(1) = 19.74, *p* < .001, with higher accuracy at the short delay than the long delay. There was no interaction between value and retention interval, χ^2^(1) = 0.85, *p* = .36.

Point-value memory performance is displayed in Table 1, as a function of value and retention interval. On point-value memory, the effect of value was not significant, χ^2^(1) = 0.76, *p* = .38. There was a main effect of retention interval, χ^2^(1) = 17.44, *p* < .001, with better memory at the short delay than the long delay. The interaction between value and retention interval was significant, χ^2^(1) = 11.16, *p* < .001, though pairwise comparisons (Bonferroni) revealed no difference between high- and low-value items at the short delay (*z* = −0.33, *p* = 1.00) or at the long delay (*z* = 1.97, *p* = .10).

### Discussion

Consistent with previous findings, Experiment 1 found that high-value items were better remembered than low-value items (Castel et al., 2002, 2007, 2011, 2013) and that memory quality (as indicated by R, K, G responses) was better in high-value items (Elliott & Brewer, 2019; Hennessee et al., 2017, 2018). Furthermore, while there was some evidence of forgetting between the different retention intervals (from 5 min to 24 hr), the effects of value remained robust and persisted over time.

Of particular interest was the effect of value on associative memory. As predicted, a memory improvement for colour information was observed for high-value items. This contrasts with previous work finding no positive effect of value on word–colour associations (Hennessee et al., 2017, 2018), indicating that value effects vary depending on the material used and the implications this has for the binding between item and associative information. In addition, although longer delay impaired colour memory overall, indicating some forgetting over time, the colour memory boost from high value was not differentially impacted, suggesting that this effect persists over time. On memory for point-value, no difference was found between high- and low-value conditions, either at the short delay or the long delay. This is consistent with previous findings (Hennessee et al., 2017) and is not unexpected as the use of coloured images might only optimise the binding between items and colours, though further work is required to confirm the reliability of this finding.

Experiment 1 established that value can positively influence item–colour associative memory under incidental encoding conditions when images are used as the stimulus set. We then moved on to examine whether word–colour associative memory might also show a value effect, when an intentional encoding condition was instead adopted.

## Experiment 2

Previous research indicates that emphasis on associative information during encoding is critical for memory performance in the binding between item and associative information. When participants were only instructed to encode item information, associative memory was poor; when they were instructed to intentionally encode both item and associative information, associative memory could be greatly improved (Chalfonte & Johnson, 1996; Hockley & Cristi, 1996; Light & Berger, 1974; Light et al., 1975). Thus, the absence of positive value effects on colour memory in previous research may reflect an inadequate integration of item and colour during encoding when word colour is encoded incidentally (Hennessee et al., 2017, 2018). Experiment 2 therefore examined whether value effects would emerge on colour memory when participants were asked to intentionally memorise both the word and its colour. With word colour an integral, explicit element in the encoding phase, we predicted that value benefits would generalise from the item to its associated colour, and therefore that both memory for words and word colour would improve for items assigned with high values. Following Experiment 1, this should be observable at both the short-delay and the long-delay tests.

### Method

#### Participants

Thirty undergraduate students (25 females; mean age = 19.80 years; range = 18–30 years) from the University of Leeds took part in the experiment. All participants were native English speakers and had correct or corrected-to-normal vision. None reported a history of neurological disorders or being colour-blind. Participants gave informed consent in accordance with the guidelines set by the University of Leeds’s Psychology Ethics Committee (Ethics reference number: PSC-462).

#### Materials and procedure

The stimuli were 176 words selected from SUBTLEXUS (Warriner et al., 2013). Each contained between three and six letters and had an everyday occurrence of at least 25 times per million according to SUBTLEXUS. Word valence ranged from 4.5 to 5.5 (scale ranges from 1 [*negative*] to 9 [*positive*]) and arousal was less than 5 (scale ranges from 1 [*calm*] to 9 [*excited*]). Half of them were randomly selected to be encoded at the study phase, with each one paired with a point-value (1 point, 10 points) and printed in one of the four colours (red, yellow, blue, green). The other half of the set was used as new items during the test phase. The procedure was the same as Experiment 1 except that participants were told to remember both the word and its colour at encoding (see Supplementary Material B for instructions).

### Results and discussion

Most of the participants completed the 24-hr delayed test within an acceptable time frame (with one night’s sleep; *N* = 21, mean time = 25 hr 11 min, range = 22 hr 31 min–31 hr 40 min). Three participants did not complete the test. Six participants took more than 2 days to complete the test (i.e., 48h17m, 52h42m, 57h46m, 65h22m, 192h54m, 211h), but including their data or not has little influence on the final results, so analyses were based on data from 27 participants. The data were analysed using GLMM. In every model, fixed factors included value, retention interval, and their interaction. In the model of item memory, random factors included random intercept and random slope of value and retention interval within participants, and random slope of value within items with intercept. The model of RKG responses has the maximal random effects structure. In the model of colour memory, random factors included random slope of value within participants with intercept, and random slope of value within items with intercept. In the model of point-value memory, random factors included random intercept and random slope of value within participants, and random intercept within items.

#### Item memory and RKG responses

Mean hit rates; false alarm rates; R, K, and G responses as a function of value, and retention interval are displayed in Table 2. On item memory, GLMM revealed a main effect of value, χ^2^(1) = 17.10, *p* < .001, with higher memory accuracy for high-value items than low-value items. The main effect of retention interval was also significant, χ^2^(1) = 6.83, *p* < .01, such that memory at the short delay was better than memory at the long delay. The interaction between value and retention interval was not significant, χ^2^(1) = 2.81, *p* = .09. On RKG responses, there was a main effect of value, χ^2^(1) = 14.83, *p* < .001, whereby memory quality was better for high-value items than low-value items. The effect of retention interval, χ^2^(1) = 0.70, *p* = .40, and the interaction between value and retention interval, χ^2^(1) = 0.09, *p* = .76, were not significant.

**Table 2.** Mean Hit Rates, False Alarm (FA) Rates, Remember (R), Know (K), Guess (G) responses and point-value memory as a function of value and retention interval in Experiment 2.

	Short delay		Long delay	
	High value	Low value	High value	Low value
Hit rates	0.47(0.03)	0.31(0.04)	0.38(0.03)	0.27(0.04)
FA rates	0.14(0.03)		0.17(0.03)	
R	0.51(0.05)	0.31(0.04)	0.49(0.04)	0.32(0.05)
K	0.22(0.03)	0.25(0.04)	0.22(0.03)	0.21(0.04)
G	0.27(0.05)	0.45(0.06)	0.28(0.04)	0.47(0.05)
Point-value	0.55(0.06)	0.37(0.05)	0.47(0.05)	0.27(0.04)

FA: false alarm.

Standard errors presented in parentheses.

#### Associative memory

**Figure 3.**
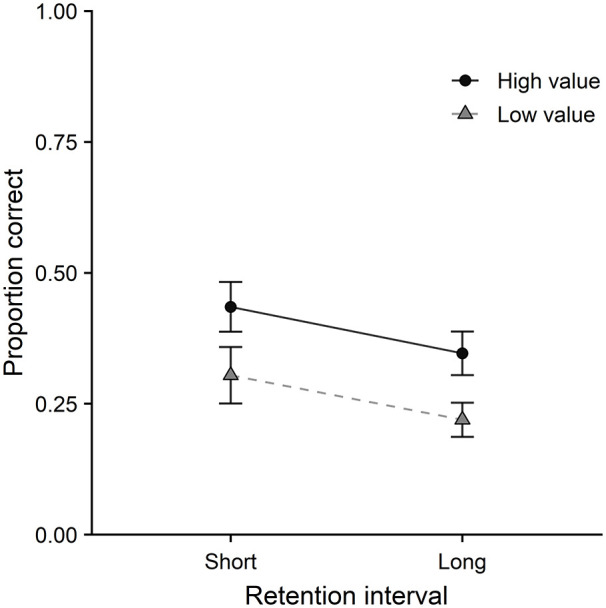
Colour memory performance as a function of value and retention interval in Experiment 2. Error bars represent one standard error of the mean.

Colour memory performance is displayed in Figure 3, as a function of value and retention interval. On colour memory, a main effect of value was observed, χ^2^(1) = 10.77, *p* = .001, with higher accuracy for high- than low-value items. A main effect of retention interval was also observed, χ^2^(1) = 17.38, *p* < .001, such that memory accuracy was higher at the short delay than the long delay. The interaction between value and retention interval was not significant, χ^2^(1) = 0.19, *p* = .67.

Point-value memory performance is displayed in Table 2, as a function of value and retention interval. On point-value memory, there was a main effect of value, χ^2^(1) = 4.80, *p* < .05, whereby memory was better for high- than low-value items. There was also a main effect of retention interval, χ^2^(1) = 30.74, *p* < .001, with better memory at the short delay than the long delay. The interaction between value and retention interval was not significant, χ^2^(1) = 0.16, *p* = .69.

The results of Experiment 2 generally replicated the value effects observed in Experiment 1, such that recognition and memory quality of high-value items were better than low-value items, across short-delay and long-delay tests. Importantly, the value effect on word colour memory was observed under intentional learning conditions, and this effect was not impaired by the passage of time. Relative to incidental colour memory encoding (Hennessee et al., 2017, 2018), word–colour binding is presumably more likely to be encoded and maintained in a durable and accessible form, and thus value effects will generalise across identity and associated colour. Likewise, memory for point-values associated with each word was also enhanced by value and was consistent across different retention intervals, though this result is inconsistent with that observed in Experiment 1.

## Experiments 3a and 3b

The final two experiments in the current series were conducted with the aim of replicating and extending previous findings regarding the value effects on word–colour binding. Thus, Experiment 3a instructed participants to remember the words but colour was incidental to the encoding phase (as in Hennessee et al. (2017)), whereas Experiment 3b instructed participants to remember both the words and the colours (as in Experiment 2 of this study). Based on previous findings, we expected to see a reliable beneficial impact of value on colour memory in Experiment 3b, but not in Experiment 3a. These final two experiments were conducted online, rather than in a lab setting (as in Experiments 1 and 2).

### Method

#### Participants

Thirty participants were recruited from Prolific (www.prolific.co; Palan & Schitter, 2018) in each experiment (Experiment 3a: 17 females, mean age = 24 years, range = 19–30 years; Experiment 3b: 14 females, mean age = 23.7 years, range = 19–30 years). All participants were native English speakers and had correct or corrected-to-normal vision. Informed consent was obtained from participants in accordance with the guidelines set by the University of Leeds’s Psychology Ethics Committee (Ethics reference number: PSYC-111).

#### Materials and procedure

The experiments were conducted online using the Gorilla Experiment Builder (www.gorilla.sc; Anwyl-Irvine et al., 2020). The materials and procedure were similar to Experiment 2. It included a study phase, a filler task, and a test phase. To maintain participant motivation and avoid attrition in an online testing environment, the experimental sessions were shortened. Sixty-four words were randomly selected from the words pool used in Experiment 2. Half of them were used as study words, the other half were used as new words during the test phase. The study words were presented in four different colours (red, yellow, blue, and green), with half paired with 1 point and the other half paired with 10 points. The study words and new words, parings between study words and point-values were counterbalanced across participants. During the study phase, each word was presented for 3 s with a 0.5-s interval. In Experiment 3a, participants were instructed that they would score either 1 point or 10 points for getting the words correct in a later memory test; in Experiment 3b, participants were instructed that they would score either 1 point or 10 points for getting the words and their colours correct in a later memory test. In both experiments, the goal was to maximise their point score. To ensure participants that maintained focus on the task during encoding, three attention-check trials were randomly presented among the study trials. Participants were instructed to press key “z” within 3 s on these trials. Following the study phase, there was a filler task (six math questions) lasted approximately 2 min. Then the old words and the new words (all in white) were presented randomly and a recognition test was conducted. For the words participants recognised as old, further RKG judgement (“Remember,” “Know,” “Guess”), colour memory test (“Red,” “Yellow,” “Blue,” “Green”) and point-value memory test (“1 point,” “10 points”) were conducted. At the end of Experiment 3a, participants were asked whether they tried to memorise the colour of the words during the study phase.

### Results

#### Experiment 3a

**Figure 4.**
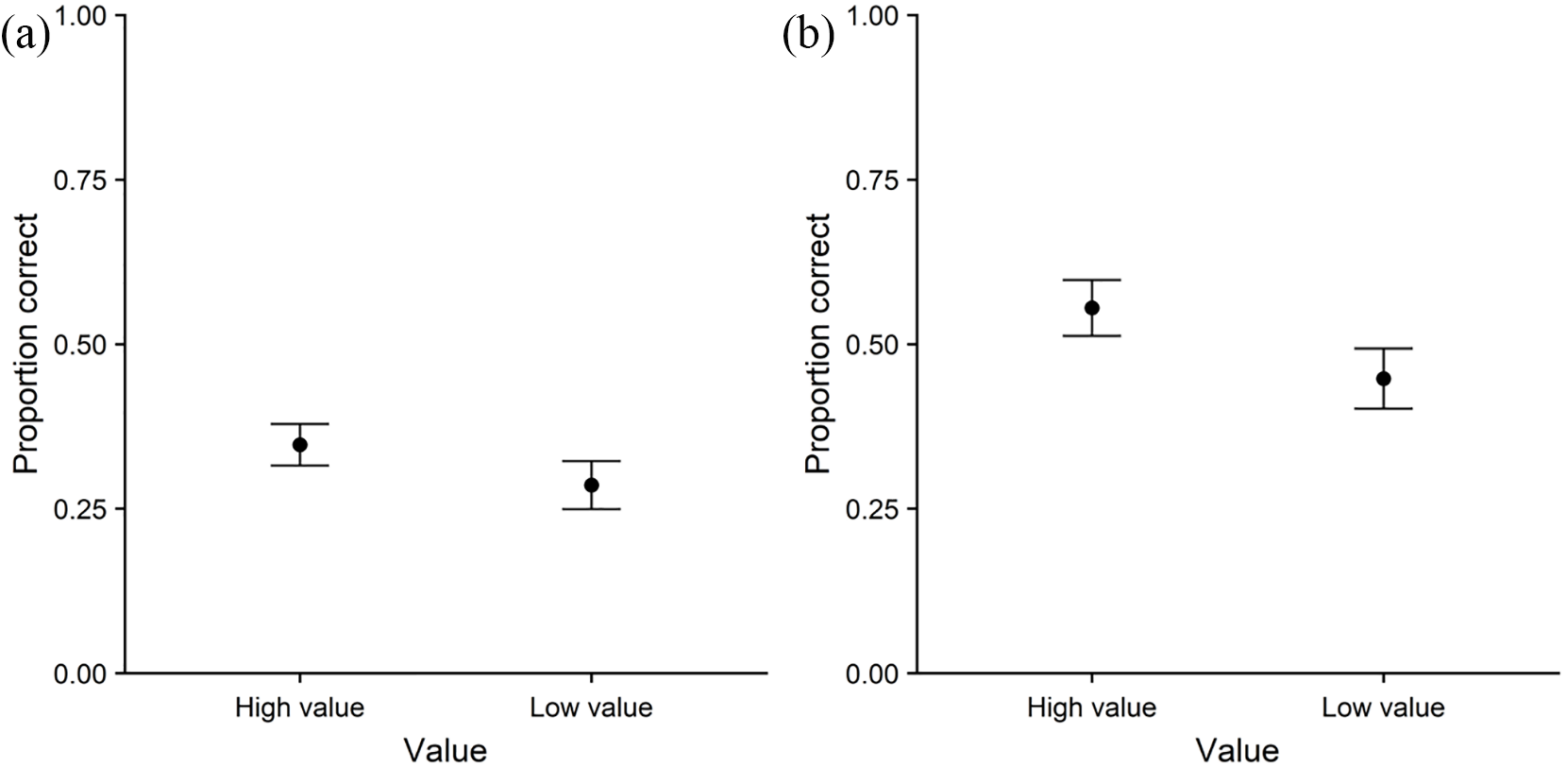
Colour memory performance as a function of value in (a) Experiment 3a and (b) Experiment 3b. Error bars represent one standard error of the mean.

Mean hit rates; false alarm rates; R, K, G responses; and point-value memory are displayed in Table 3, as a function of value. Colour memory is displayed in Figure 4a, as a function of value. Item memory, colour memory, and point-value memory were analysed using GLMM; RKG responses was analysed using CLMM. In each model, the fixed factor was value. In the model of item memory, the random factors included random intercept and random slope of value within participants, and random intercept within items. In the models of RKG responses and point-value memory, random factors included random intercept and random slope of value within participants, and within items. In the model of colour memory, random factors included random intercept and random slope of value within items, and random intercept within participants. On item memory, GLMM revealed a main effect of value, χ^2^(1) = 18.58, *p* < .001, such that memory was better for high-value items than low-value items. On RKG responses, the main effect of value was also significant, χ^2^(1) = 4.57, *p* < .05, with better memory quality for high- than low-value items. On colour memory, no effect of value was observed, χ^2^(1) = 1.53, *p* = .22. Nine participants reported that they memorised the colours intentionally during the study phase. Removing their data revealed similar result, χ^2^(1) = 1.06, *p* = .30. On point-value memory, the effect of value was also not significant, χ^2^(1) = 0.24, *p* = .63.

**Table 3.** Mean Hit Rates, False Alarm (FA) Rates, Remember (R), Know (K), Guess (G) responses and point-value memory as a function of value and retention interval in Experiment 3a and Experiment 3b.

	Experiment 3a		Experiment 3b	
	High value	Low value	High value	Low value
Hit rates	0.71(0.04)	0.50(0.04)	0.48(0.03)	0.42(0.03)
FA rates	0.15(0.03)		0.12(0.02)	
R	0.40(0.06)	0.33(0.05)	0.41(0.05)	0.29(0.05)
K	0.42(0.05)	0.39(0.05)	0.34(0.04)	0.35(0.04)
G	0.18(0.03)	0.29(0.04)	0.25(0.04)	0.36(0.05)
Point-value	0.64(0.04)	0.67(0.05)	0.52(0.05)	0.65(0.05)

FA: false alarm.

Standard errors presented in parentheses.

#### Experiment 3b

Mean hit rates; false alarm rates; R, K, G responses; and point-value memory are displayed in Table 3, as a function of value. Colour memory is displayed in Figure 4b, as a function of value. Value was the fixed factor in each model. In the model of item memory, the random factors included random intercept and random slope of value within participants, and random intercept within items. In the models of RKG responses and point-value memory, the random factors included random intercept and random slope of value within participants. In the model of colour memory, the random factors included random intercept within participants, and random intercept within items. GLMM revealed no effect of value on item memory, χ^2^(1) = 2.63, *p* = .11. On RKG responses, there was a main effect of value, χ^2^(1) = 6.76, *p* < .01, such that high-value items were associated with better memory quality. The effect of value was significant on colour memory, χ^2^(1) = 7.41, *p* < .01, but not significant on point-value memory, χ^2^(1) = 2.20, *p* = .14.

### Discussion

The aim of Experiments 3a and 3b was to replicate the outcomes of Experiment 2 regarding value effects on intentional word–colour memory associations, while also demonstrating that such effects are much less reliable when using incidental colour encoding as found in previous studies (Hennessee et al., 2017, 2018). In Experiment 3a, when participants were instructed to remember words (but colour was incidental), we found a value effect on item memory but there was no significant effect on colour memory, in line with previous studies in the area. In Experiment 3b, when participants were instructed to remember both the words and colours, the value effect on colour memory re-emerged. These results verified that when the binding condition between item and colour is optimised, the influence of value could extend to colour information. On point-value memory, no value effect was observed from either experiment. These results are consistent with Experiment 1 rather than Experiment 2, suggesting the effect of value on point-value memory is somewhat unreliable.

Although the focus of this study was on associative rather than item memory, it is worth noting that the value effect on item memory was not observed in Experiment 3b (in contrast to Experiment 2). Speculatively, one possibility could be that online participants may have invested less energy and concentration in the task than those involved in a laboratory experiment (Kraut et al., 2004), and thus may have been less likely to use deeper strategic encoding, likely an important mechanism underlying the value effect on item memory (Cohen et al., 2014; Hennessee et al., 2019). However, we have no direct evidence to support this suggestion at present; it would be valuable for future work to carefully explore the extent to which value effects emerge for both item and associative memory across different levels of manipulations such as participant engagement, attentional load, and strategic approach.

## General discussion

Across four experiments, this study explored whether value enhances memory for associative information under different conditions in which the binding between item information and associative information is optimised, and whether this memory enhancement effect persists over time. Using coloured images (Experiment 1) and intentional learning of word colour (Experiment 2), it was consistently found that value improved memory for colour information and this effect persisted over a longer delay (approximately 24 hr). Experiments 3a (incidental word colour) and 3b (intentional word colour) focused on memory over short delays and successfully replicated the main outcomes of Experiment 2 and of previous studies in the area. Alongside these key novel findings, this study also replicated previous findings that item recognition and memory quality were superior in high-value items, relative to low-value items (Castel et al., 2002, 2013; Hennessee et al., 2017, 2018), and extended these observations over longer periods of time.

How might we explain the memory enhancement effects of value that were observed? First, it is possible that high-value items are allocated with more attentional resources during encoding (Allen, 2019; Miller et al., 2019). Within the context of working memory (Hu et al., 2016) or LTM (Elliott & Brewer, 2019), the memory advantage for high-value items has been shown to reduce as a result of concurrent divided attention, although other studies have found that such tasks only impair overall memory and do not reduce value-directed prioritisation effects (Atkinson et al., 2020; Middlebrooks et al., 2017; Siegel & Castel, 2018a, 2018b). Nevertheless, when participants are given the choice to decide what information to study and how to study it, they spend more time studying and restudying the high-value items, relative to low-value items (Castel et al., 2013; Middlebrooks & Castel, 2018; Robison & Unsworth, 2017). Similarly, Miller et al. (2019) used pupillometry as an index of attention and observed increased pupillary responses during encoding of items at high- relative to low-value serial positions. Thus, more attentional resources may be allocated to the encoding of high-value items.

A second, related, possibility is that high-value items are engaged with via deeper strategic encoding. Hennessee et al. (2019) found that instructing participants to use sentence generation and mental imagery strategies across both high- and low-value conditions eliminated/nearly eliminated value effects on recognition, suggesting this effect is due to more elaborative encoding strategies for high-value items. Similarly, Bui et al. (2013) showed that enhanced relational processing among high-value items is a possible mechanism underlying the value effects. These findings are consistent with participants’ self-report that they use more effective strategies (i.e., imagery mediators, keyword mediators, sentence generation, or relational processing) when learning high-value word pairs (Ariel et al., 2015). Thus, in the context of this study, valuable item–colour bindings may be engaged with using strategic encoding techniques such as subvocal rehearsal (e.g., mentally repeat “red iron”) and associating items with colours (e.g., the iron is red because it is hot). Third, it may also involve a (possibly dopaminergic) memory consolidation process (Murayama & Kitagami, 2014; Murayama & Kuhbandner, 2011; Spaniol et al., 2013). Reward-related motivation is thought to activate the dopaminergic midbrain and the hippocampus (Shohamy & Adcock, 2010), and this in turn enhances hippocampal-dependent memory consolidation (Wittmann et al., 2005).

When considered in the context of prior work examining value effects on associative memory (Hennessee et al., 2017, 2018), the conditions for binding between item information and colour information that were implemented in this study appear to have optimised the likelihood of value effects generalising across item identity and colour. One potential reason is that the specific binding conditions implemented in a task help determine whether associative information is initially registered and maintained, possibly within the focus of attention (FoA) within working memory (see e.g., Cowan, 1999; Hitch et al., 2020). Further encoding processes, for example, continued attentional and/or strategic processing, would then be implemented according to value, thus giving rise to memory benefits for item and associative information. Thus, in Experiment 1, the use of conjunctive bindings within which colour information is an integral part of each image may have resulted in colour being more likely to be encoded into and maintained within the FoA. This is consistent with the object file theory that attention to any one property of an object causes other properties of that object to be attended (Kahneman et al., 1992; Treisman & Zhang, 2006). In Experiment 2 and Experiment 3b, colour information was maintained in the FoA through a form of relational binding based on the intentional learning of words and colours. In Experiment 3a and previous studies (Hennessee et al., 2017, 2018), however, colour information might not have been the maintained in the FoA through incidental learning of word colour, thus no value effect was observed on colour memory. In line with this explanation, previous positive findings regarding value enhancement effects on associative memory may reflect associative information being entered into the FoA at encoding via intentional learning, such as memory for visuospatial bindings (Siegel & Castel, 2018a, 2018b), memory for word pairs (Ariel et al., 2015), and memory for word plurality status (Cohen et al., 2017).

These value effects persist more than 24 hr, indicating that rather than being transient, they are potentially robust and long-lasting. There was no evidence of that such effects increased in size, as observed in previous studies (e.g., Murayama & Kuhbandner, 2011; Spaniol et al., 2013). Among various methodological differences, there was no monetary value attached to our items, which might be an important factor in engaging enhanced dopaminergic consolidation over time. In addition, it should be noted that, in this study, all items were tested at both the short- and long-delay test points. As literature on the testing effect indicates memory can be enhanced through testing and retrieval (e.g., Karpicke et al., 2007; Roediger et al., 2006), value effects at the longer delay may at least partly reflect their more successful retrieval at the earlier test point. Nevertheless, it is clear that the effect of value, both on item memory and on colour memory persists after a 24-hr delay. Future studies should systematically explore the longevity of the value effect and how it might interact with intervening bouts of testing and retrieval.

Results regarding point-value memory are inconsistent in the current experiments. There was a value effect in Experiment 2, but it was not observed in Experiment 1, 3a, or 3b. Indeed, previous findings regarding the value effect on point-value memory have also been inconsistent (Hennessee et al., 2017, 2018). Point-values inform how the participant approaches each item during the encoding phase, thus there may be a relatively weak incidental binding formed between each item and its value but this does not always reliably survive to the test phase. It could be useful for future work to explore whether value effects on point memory also emerge when this is made an explicit part of the encoding phase, and whether this then impacts on other value effects that are observed. Indeed, it is useful to note that colour memory improved for high-value items in Experiments 1 and 3b, even though participants were not reliably better at retrieving the associated values of these items. This supports the idea that value influences colour memory at least in part during the encoding phase.

One methodological difference between the current experiments and previous studies (Hennessee et al., 2017, 2018) that may be worth noting relates to the variation in the number of different point values that are allocated to items. The current experiments adopted the approach used in exploration of value effects in working memory (see Hitch et al., 2020) and applied a binary high-low distinction (i.e., 1 point for low value and 10 points for high value), whereas there were six different point values (i.e., 1, 2, and 3 for low value and 10, 11, and 12 for high value) in Hennessee et al. (2017). Value effects for shape–colour binding have been found in a working memory context using a continuous rather than a dichotomous high-low value system (Hu et al., 2014). Nevertheless, the dichotomous value structure used in this study may be easier for participants to distinguish between high- and low-value items and reduce the complexity of the taskset, which may enable a more effective focus on high-value items. Consistent with this idea, Villaseñor et al. (2021) found a value effect on a subjective (though not an objective) measure of context memory when the range of point values were reduced from 1 to 8 to 1 to 4. Thus, although Experiments 3a (incidental colour encoding) and 3b (intentional colour encoding) replicated the relative pattern of findings from our Experiment 2 and previous studies using a binary value system, it would be worthwhile for future studies to explore the extent to which variability and complexity of value allocation might impact on changes in value effects.

In conclusion, across four experiments examining different types of binding condition, this study shows that memory for associative information can indeed be improved when items are allocated with increased value. Thus, value effects can be observed from item recognition, quality of memory, and associative memory. Research should continue to explore the mechanisms underlying value-directed remembering effects across different tasks contexts and time frames, and the implications of this for optimising memory efficiency.

## Supplemental Material

sj-docx-1-qjp-10.1177_17470218211014439 – Supplemental material for The effect of value on long-term associative memory Click here for additional data file. Supplemental material, sj-docx-1-qjp-10.1177_17470218211014439 for The effect of value on long-term associative memory by Xiaotong Yin, Jelena Havelka and Richard J Allen in Quarterly Journal of Experimental Psychology
