# Supplementary material for: The effect of value on long-term associative memory
Source: Q J Exp Psychol (Hove). 2021 May 15;74(12):2033–45. doi: 10.1177/17470218211014439 (PMC8531949; doi:10.1177/17470218211014439)
Supplement: sj-docx-1-qjp-10.1177_17470218211014439 – Supplemental material for The effect of value on long-term associative memory [file sj-docx-1-qjp-10.1177_17470218211014439.docx]

Supplementary Material for:

**The Effect of Value on Long-Term Associative Memory**

Xiaotong Yin, Jelena Havelka, and Richard J. Allen

**A. Instructions in Experiment 1**

**Study phase instructions**

Welcome to our experiment! This session will last around 30 minutes. You will be presented with a series of images. Each of them is associated with a point-value (1 point or 10 points) which you could earn later for recognition. Each image will be shown for 3 seconds. Your task is to try to remember the images and to maximize your points score. Following this presentation phase, there will then be a brief calculation task lasting a few minutes. After this, you will be shown a mixture of the images you saw earlier and some new images, and a recognition test will be conducted.

**Remember-know-Guess instructions**

***Remember:***

When you see an image, if it triggers something that you experienced when you saw it previously, like, for example, something about its appearance on the screen or the order in which the image came in, please indicate this kind of recognition, by choosing the REMEMBER option. In other instances the image may remind you of something you thought about when you saw it previously, like an association that you made to the image, or something of personal significance that you associated with the image; again if you can recollect any of these aspects of when the image was first presented please choose the REMEMBER option.

***Know:***

At other times you will see an image and you will recognize it as one you saw previously, but the image will not bring back to mind anything you remember about seeing it then. When you feel confident that you saw the image previously, even though you do not recollect anything you experienced when you saw it, please choose the KNOW option. With KNOW responses you are sure about seeing the image previously but cannot remember the circumstances in which the image was presented, or the thoughts elicited when the image was presented.

***Guess:***

With a GUESS response, you think it possible that the image was presented but you are not sure that it was. For some reason, you think there was a chance that the image was presented. Some people say ‘‘it looks like one of those images that could possibly have been there.’’ When you think your response was really just a guess, please choose the GUESS option.

**B. Instructions in Experiment 2**

**Study phase instructions**

Welcome to our experiment! This session will last around 30 minutes. You will be presented with a series of words in different colours (red, yellow, blue and green), each associated with a point-value (1 point or 10 points) you could earn later for recognition. Each word will be shown for 3 seconds. Your task is to try to remember the words and their colours and to maximize your points score. Following this presentation phase, there will then be a brief calculation task lasting a few minutes. After this, you will be shown a mixture of the words you saw earlier and some new words, and a recognition test will be conducted.

**Remember-know-Guess instructions**

***Remember:***

When you see a word, if it triggers something that you experienced when you saw it previously, like, for example, something about its appearance on the screen or the order in which the word came in, please indicate this kind of recognition, by choosing the REMEMBER option. In other instances the word may remind you of something you thought about when you saw it previously, like an association that you made to the word, or an image that you formed when you saw the word, or something of personal significance that you associated with the word; again if you can recollect any of these aspects of when the word was first presented please choose the REMEMBER option.

***Know:***

At other times you will see a word and you will recognize it as one you saw previously, but the word will not bring back to mind anything you remember about seeing it then. When you feel confident that you saw the word previously, even though you do not recollect anything you experienced when you saw it, please choose the KNOW option. With KNOW responses you are sure about seeing the word previously but cannot remember the circumstances in which the word was presented, or the thoughts elicited when the word was presented.

***Guess:***

With a GUESS response, you think it possible that the word was presented but you are not sure that it was. For some reason, you think there was a chance that the word was presented. Some people say ‘‘it looks like one of those words that could possibly have been there.’’ When you think your response was really just a guess, please choose the GUESS option.
